# Impact of banning smoking in cars with children on exposure to second-hand smoke: a natural experiment in England and Scotland

**DOI:** 10.1136/thoraxjnl-2019-213998

**Published:** 2020-01-27

**Authors:** Anthony A Laverty, Thomas Hone, Eszter P Vamos, Philip E Anyanwu, David Taylor-Robinson, Frank de Vocht, Christopher Millett, Nicholas S Hopkinson

**Affiliations:** 1 Public Health Policy Evaluation Unit, School of Public Health, Imperial College London, London, UK; 2 Global Digital Health Unit, Department of Primary Care and Public Health, Imperial College London, London, UK; 3 Department of Public Health and Policy, University of Liverpool, Liverpool, UK; 4 Population Health Sciences, Bristol Medical School, University of Bristol, Bristol, UK; 5 National Heart and Lung Institute, Imperial College London, London, UK

**Keywords:** smoking cessation, tobacco and the lung

## Abstract

England banned smoking in cars carrying children in 2015 and Scotland in 2016. We used survey data from 3 years for both countries (N_England_=3483–6920, N_Scotland_=232–319) to assess effects of the English ban using logistic regression within a difference-in-differences framework. Among children aged 13–15 years, self-reported levels of regular exposure to smoke in cars for Scotland were 3.4% in 2012, 2.2% in 2014 and 1.3% in 2016 and for England 6.3%, 5.9% and 1.6%. The ban in England was associated with a −4.1% (95% CI −4.9% to −3.3%) absolute reduction (72% relative reduction) in exposure to tobacco smoke among children.

## Introduction

Exposure to secondhand tobacco smoke is a significant cause of illness in children and particularly affects more disadvantaged groups.^1^ Exposure of children to smoking inside cars is especially concerning owing to the very high concentrations of smoke in enclosed spaces and associations with child smoking uptake.^2 3^ Both smoking uptake and secondhand exposure to smoke are mechanisms which sustain health inequality.^4–6^

One policy response has been bans on smoking in private vehicles with children present. Evaluations of such bans are scarce and present a mixed picture.^7–9^ A ban on smoking in private vehicles with anyone aged ≤18 years present came into effect on 1 October 2015 in England, and on 5 December 2016 in Scotland, providing a natural experiment to evaluate policy effects.

## Methods

Data for England came from the Smoking, Drinking and Drug Use (SDDU) surveys and for Scotland from the Scottish health surveys in 2012, 2014 and 2016 (online supplementary appendix 1). We restricted the sample to children aged 13–15, as exposure for 11 and 12 year olds was reported by caregivers, which is likely to lead to under-recording.

10.1136/thoraxjnl-2019-213998.supp1 Supplementary data

Our primary exposure was child-reported regular exposure to smoking inside cars, assessed, in England, with the question “In the past year, how often were you in a car with somebody smoking?” (table 1). We categorised responses of *every day or most days* as 'regular exposure'. In Scotland, children were asked “Are you regularly exposed to other people’s tobacco smoke in any of these places?” (Responses: yes/no for cars/vehicles). We harmonised a measure of deprivation using free school meals and the Family Affluence Scale and include this as well as age and sex in analyses (table 1).

**Table 1:** Key characteristics of data sources

Country	England	Scotland
Data name	Smoking Drinking Drug Use (SDDU) survey	Scottish health survey
Years and interview dates	**2012:** September 2012 – December 2012 **2014:** September 2014 – December 2014 **2016:** September 2016 – January 2017	**2012:** January 2012 – December 2012 **2014:** January 2014 – February 2015 **2016:** January 2016 – January 2017
Individuals included	**2012:** 4915 **2014:** 3483 **2016:** 6920	**2012:** 319 **2014:** 271 **2016:** 232
Age range	11–15 years	13–17 years
Exposure to tobacco smoke in cars question	**2012:** Two separate questions: “*In the past year, how often were you in your family’s car (* *OR* *) someone else’s car with somebody smoking?” (Responses: every day or most days; once or twice a week; once or twice a month; less often than once a month; never in the past year; don’t know*) **2014 and 2016:** In the past year, how often were you in a car with somebody smoking? This could be your family’s car or someone else’s car. (*Responses: every day or most days; once or twice a week; once or twice a month; less often than once a month; never in the past year; don’t know*) In this study ‘*every day or most days’* classed as 'regular exposure'	**2012–2016:** “Are you regularly exposed to other people’s tobacco smoke in any of these places?” (responses: yes/no for in cars/vehicles, etc)
Deprivation marker	**2012 and 2014:** Child reported receipt of free school meals (FSM)	**2012– 2016:** Scottish index of multiple deprivation in five groups
	**2016:**Family Affluence Scale (low, middle, high). Harmonised so low groupe quivalent to receiving FSM	Harmonised so most deprived group comparable to receiving FSM or being in lowest affluence band

We used survey-weighted logistic regression to assess changes in exposure over time using a differences-in-differences analysis, which controls for all time-invariant differences between the intervention and comparison populations.^10^ We considered interaction between time and country to assess changes in each country separately and with policy implementation in England in 2016. We present relative percentage changes (based on odds ratios) and absolute percentage changes (average marginal effects compared with baseline).

We conducted various additional analyses: an unadjusted model (online supplementary appendix table 2), analyses with linear trends for each country (online supplementary appendix table 3) and analyses for England only (ages 11–15), using more granular exposure data to analyse ever, monthly and regular exposure (online supplementary appendix table 4).

## Results

**Figure 1:**
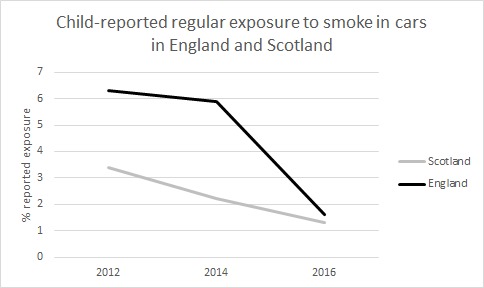
Percentages of child-reported regular exposure to smoke in cars in England and Scotland 2012–2016

A total of 15 318 responses were received in England and 822 in Scotland (online supplementary appendix 1). Self-reported regular exposure to smoke in cars was 6.3% in 2012, 5.9% in 2014 and 1.6% in 2016 for England and, respectively, 3.4%, 2.2% and 1.3% in Scotland (figure 1).

Implementation of the smoke-free policy in England was associated with an absolute reduction of −4.1% (95% CI −4.9% to −3.3%) (72% relative reduction) in the percentage of children self-reporting exposure to smoke in cars (table 2). Girls and those in the deprived group were more likely to report exposure.

**Table 2:** Results from logistic regression difference in difference analyses of impact of policy implementation on self-reported exposure to smoking in vehicles

	AOR (95% CI)	Absolute % difference (95% CI)
Scotland 2012–2014	0.34 (0.15 to 0.80)	−0.45 (-2.70 to 1.80)
Scotland 2014–2016	0.25 (0.07 to 0.79)	0.00 (-1.05 to 1.06)
England 2012–2014	1.00 (0.82 to 1.22)	−1.04 (-3.34 to 1.27)
England 2014–2016 (policy implementation)	0.28 (0.21 to 0.37)	−4.11 (-4.91 to −3.31)
Age 13 years	ref	ref
Age 14 years	1.01 (0.80 to 1.28)	0.04 (-0.77 to 0.84)
Age 15 years	1.22 (0.99 to 1.51)	0.74 (-0.05 to 1.53)
Boys	ref	ref
Girls	1.61 (1.34 to 1.93)	1.75 (1.07 to 2.43)
Not deprived group	ref	ref
Deprived group	1.98 (0.82 to 1.21)	2.53 (1.76 to 3.29)

Absolute % differences derived from marginal effect compared with baseline.

AOR, adjusted odds ratio.

Analyses unadjusted for covariates and using linear time trends were similar (online supplementary appendix tables 2 and 3). Analyses within England only, using the wider age range of 11 to 15 years, identified substantial changes in exposure in all levels of exposure assessed after implementation of the ban (online supplementary appendix table 4).

## Discussion

Child-reported frequency of exposure to tobacco smoke in cars fell after the 2015 introduction of the ban in England, a finding made more robust by comparison with Scotland where the policy was not introduced until the following year. This exposure remains higher in children from more deprived groups, which serves as a reminder of the socially patterned risks of smoking. These findings provide support for introducing this policy in other jurisdictions, although further evaluations are warranted. Previous research from Canadian provinces enacting such bans found more marked effects on exposure in provinces with comprehensive strategies including discouraging smoking uptake, while recent evidence has additionally pointed to exposure to smoking in cars resulting in an increase in the incidence of asthma.^8 9^

The ban is an example of the 'Protect' element of the MPOWER policy approach to delivering the WHO Framework Convention on Tobacco Control programme to reduce the harms caused by smoking. Importantly, the purpose of the ban is to reduce child exposure to tobacco smoke, for which this study provides evidence, and not to drive prosecutions (eg, coverage presenting the legislation as a failure due to the low number of arrests https://www.mirror.co.uk/news/uk-news/car-smoking-ban-massive-flop-10858407).

### Strengths and limitations

The strength of this experiment using high-quality data from Scotland and England is that the design permits observed changes to be plausibly ascribed to the policy intervention. However, differences in the data used between countries deserve consideration. Different measurements of exposure in England and Scotland mean that we cannot directly compare exposure levels between countries, and the measure of deprivation we use has been harmonised from measures which have not previously been combined. However, analyses of data for England alone, using different levels of exposure, and not including the deprivation measure, all produced concordant results. Combining data sources also meant that we could employ a difference-in-difference design, which controls for time-invariant differences between countries and provides stronger evidence than relying on England alone. Exposure was based on self-report only, and reporting bias might have changed over time, although this would probably have been similar in both countries. Interview dates were not available for the Scottish data, and sampling in 2016 included almost 2 months after the introduction of their ban. Misclassification from sampling dates would probably bias associations towards the null point, but the direction of any biases due to harmonising data sources is difficult to predict. Reported frequencies of exposure frequency in Scotland were low, meaning that there might have been some floor effects and, as these analyses use only three data points from each country, future analyses with more data are recommended and may provide discrepant results.

## Conclusions

Our results suggest that banning smoking in private vehicles carrying children has been successful in its main aim of reducing their exposure to tobacco smoke. Given children’s known vulnerability to secondhand smoke, reductions in exposure will probably result in improved health.
