# Supplementary material for: Impact of banning smoking in cars with children on exposure to second-hand smoke: a natural experiment in England and Scotland
Source: Thorax. 2020 Jan 27;75(4):345–7. doi: 10.1136/thoraxjnl-2019-213998 (PMC7231450; doi:10.1136/thoraxjnl-2019-213998)
Supplement: Supplementary data [file thoraxjnl-2019-213998supp001.pdf]

Impact of banning smoking in cars with children on exposure to second-hand smoke: a natural experiment in England and Scotland - APPENDIX

Anthony A Laverty[1*], Thomas Hone[1], Philip E. Anyanwu [2], David Taylor Robinson[3], Frank de Vocht[4], Christopher Millett[1], Nicholas S Hopkinson[5]

[1] Public Health Policy Evaluation Unit, School of Public Health, Imperial College London, UK

[2] Global Digital Health Unit, School of Public Health, Imperial College London, London, UK

[3] Centre for Public Health, Bristol Medical School, University of Bristol, Bristol, UK

[4] Department of Public Health and Policy, University of Liverpool, Liverpool, England

[5] National Heart and Lung Institute, Imperial College London, London, UK

* Room 322, Reynolds Building, Imperial College London, St Dunstan's Road, W6 8RP, London, England.

Appendix contents

1. Further information on data sources
2. Appendix table 1 of study sample
3. Appendix table 2 of analyses using linear trends for time
4. Appendix table 3 unadjusted analyses
5. Appendix table 4 of analyses in England only including children aged 11 – 15 years

Further information on data sources

The Smoking Drinking Drug Use (SDDU) is a survey of children in school years 7 – 11 (aged 11-15 years) and is used to monitor the performance of the Government tobacco strategy [1][2][3][4][5].Data came from questionnaires administered to children at school in exam conditions and is designed to be representative of the gender, age, region and type of school in England. Data for Scotland come from the years 2012, 2014 and 2016, where children aged 13 years and older were asked to report their exposure to smoking in cars [6][7][8]. For children below this age, caregivers were asked to report exposure, but we have excluded this data due to concerns over the accuracy of caregiver reporting of exposure.

Both surveys use a using a multi-stage stratified design and we have applied survey weights as recommended in the reports from both the SDDU and the Scottish health survey. Both surveys recommend the use of one weighting variable for analyses. The SDDU recommends the use of one weighting variable (*pupilwt)* which accounts for unequal selection probabilities between regions of England, sex and school year [9]. The Scottish Health Survey also recommends the use of one survey weight in analyses of children (*cmint[year]wt).* This weight is designed to adjust for selection of only two children per household as well as differences between responding and non-responding households. These survey weights were used for all regression analyses, and analyses were conducted in Stata using the survey routine *svy: logistic* There were differences in the collection of data on markers of deprivation over time in England. In 2012 and 2014 children were asked if they received Free School Meals (FSM), but this measure was not used in 2016. The 2016 data used the Family Affluence Scale which asks children how many computers, vehicles and bathrooms their family has and assigned them a band from low to high [10]. We have harmonised these two measures by considered those receiving FSM or in the low FAS band as deprived. Scottish data used the Scottish Index of Multiple Deprivation as a marker of deprivation, and we harmonised the data by using the most deprived group as equivalent to receiving FSM or being in the lowest FAS band.

Appendix table 1: Study sample

	**England**		**Scotland**	
	**Overall N**	**% exposed**	**Overall N**	**% exposed**
2012	4915	6.3	319	3.4
2014	3483	5.9	271	2.2
2016	6920	1.6	232	1.3
Age 13 years	5056	4.0	272	0.7
Age 14 years	4702	3.8	266	3.4
Age 15 years	5560	4.5	284	3.2
Boys	7617	3.3	429	1.4
Girls	7701	4.8	393	3.6
Not deprived group	13572	3.5	552	2.2
Deprived group	1746	8.8	270	3.0
Total	15318	4.1	822	2.4

Appendix Table 2: Results from analyses using linear trends for time

	AOR	Lower CI	Upper CI
Policy Implementation	0.27	0.19	0.41
Scotland trend	0.77	0.42	1.42
England Trend	1.01	0.82	1.21
Scotland baseline	ref	ref	ref
England baseline	2.35	1.28	4.30
Age 13 years	ref	ref	ref
Age 14 years	1.01	0.80	1.28
Age 15 years	1.22	0.99	1.51
Boys	ref	ref	ref
Girls	1.61	1.34	1.93
Not deprived group	ref	ref	ref
Deprived group	1.98	1.61	2.43

AOR = Adjusted Odds Ratio, CI = Confidence Interval

In these analyses we modelled a linear trend for time individually for both Scotland and England individually and a binary variable for 2016 in England as the one post-intervention data point, interacted with time.

Results as odds ratios from logistic regression

Appendix table 3 : Results from unadjusted analyses

	Absolute % difference	Lower CI	Upper CI
Scotland 2012 - 2014	-0.66	-3.35	2.03
Scotland 2014-2016	0.02	-1.06	1.09
England 2012 - 2014	-1.55	-4.14	1.03
England 2014 - 2016 (policy implementation)	-4.14	-4.94	-3.33

Appendix table 4: Difference in difference analyses in England only including children aged 11 – 15 years

	Absolute % difference	Lower CI	Upper CI
Regular exposure			
Change 2012 - 2014	-2.96	-4.87	-1.05
Change 2014 - 2016	-10.83	-12.99	-8.67
Monthly exposure			
Change 2012 - 2014	-3.93	-5.59	-2.26
Change 2014 - 2016	-11.33	-13.09	-9.56
Ever exposure			
Change 2012 - 2014	0.17	-0.66	1.01
Change 2014 - 2016	-3.86	-4.48	-3.24

These analyses use data from England only and are controlled for age (11 – 15 years, categorically), sex and a marker of deprivation and survey weights for non-response.

References

1 NHS Digital. Smoking, Drinking and Drug Use Among Young People in England. 2016.

2 Department of Health (2017) Towards a Smokefree Generation: A Tobacco Control Plan for England. https://assets.publishing.service.gov.uk/government/uploads/system/uploads/attachment_data/file/630217/Towards_a_Smoke_free_Generation_-_A_Tobacco_Control_Plan_for_England_2017-2022__2_.pdf

3 National Centre for Social Research, National Foundation for Educational Research. (2013). Smoking, Drinking and Drug Use among Young People, 2012. [data collection]. UK Data Service. SN: 7393, http://doi.org/10.5255/UKDA-SN-7393-1.

4 NatCen Social Research. (2015). Smoking, Drinking and Drug Use among Young People, 2014. [data collection]. UK Data Service. SN: 7811, http://doi.org/10.5255/UKDA-SN-7811-1.

5 NHS Digital. (2018). Smoking, Drinking and Drug Use among Young People, 2016. [data collection]. UK Data Service. SN: 8320, http://doi.org/10.5255/UKDA-SN-8320-1.

6 ScotCen Social Research, University College London, Department of Epidemiology and Public Health, University of Glasgow, MRC/CSO Social and Public Health Sciences Unit. (2015). Scottish Health Survey, 2012. [data collection]. 3rd Edition. UK Data Service.

7 ScotCen Social Research. (2019). Scottish Health Survey, 2014. [data collection]. 3rd Edition. UK Data Service. SN: 7851, http://doi.org/10.5255/UKDA-SN-7851-3.

8 ScotCen Social Research. (2017). Scottish Health Survey, 2016. [data collection]. UK Data Service. SN: 8290, http://doi.org/10.5255/UKDA-SN-8290-1.

9 NHS England (2017). Smoking, Drinking and Drug Use among Young People in England 2016 Appendices https://files.digital.nhs.uk/publication/j/b/sdd-2016-app1.pdf.

10 Currie C, Molcho M, Boyce W, *et al.* Researching health inequalities in adolescents: The development of the Health Behaviour in School-Aged Children (HBSC) Family Affluence Scale. *Soc Sci Med* Published Online First: 2008. doi:10.1016/j.socscimed.2007.11.024
